# Predictors of surgical site infection following reconstructive flap surgery: A multi-institutional analysis of 37,177 patients

**DOI:** 10.3389/fsurg.2023.1080143

**Published:** 2023-01-30

**Authors:** Bashar Hassan, Abdulghani Abou Koura, Adham Makarem, Kamal Abi Mosleh, Hani Dimassi, Hani Tamim, Amir Ibrahim

**Affiliations:** ^1^ Scholars in HeAlth Research Program (SHARP), American University of Beirut Medical Center, Beirut, Lebanon; ^2^ School of Pharmacy, Lebanese American University, Byblos, Lebanon; ^3^ Clinical Research Institute, Faculty of Medicine, American University of Beirut, Beirut, Lebanon; ^4^ College of Medicine, Alfaisal University, Riyadh, Saudi Arabia; ^5^ Division of Plastic and Reconstructive Surgery, American University of Beirut Medical Center, Beirut, Lebanon

**Keywords:** surgical site infection, SSI, reconstructive flap surgery, reconstructive surgery, flaps, predictors, risk factors, NSQIP

## Abstract

**Purpose:**

Rates of surgical site infection (SSI) following reconstructive flap surgeries (RFS) vary according to flap recipient site, potentially leading to flap failure. This is the largest study to determine predictors of SSI following RFS across recipient sites.

**Methods:**

The National Surgical Quality Improvement Program database was queried for patients undergoing any flap procedure from years 2005 to 2020. RFS involving grafts, skin flaps, or flaps with unknown recipient site were excluded. Patients were stratified according to recipient site: breast, trunk, head and neck (H&N), upper and lower extremities (UE&LE). The primary outcome was the incidence of SSI within 30 days following surgery. Descriptive statistics were calculated. Bivariate analysis and multivariate logistic regression were performed to determine predictors of SSI following RFS.

**Results:**

37,177 patients underwent RFS, of whom 7.5% (*n *= 2,776) developed SSI. A significantly greater proportion of patients who underwent LE (*n *= 318, 10.7%) and trunk (*n *= 1,091, 10.4%) reconstruction developed SSI compared to those who underwent breast (*n *= 1,201, 6.3%), UE (*n *= 32, 4.4%), and H&N (*n *= 100, 4.2%) reconstruction (*p* < .001). Longer operating times were significant predictors of SSI following RFS across all sites. The strongest predictors of SSI were presence of open wound following trunk and H&N reconstruction [adjusted odds ratio (aOR) 95% confidence interval (CI) 1.82 (1.57–2.11) and 1.75 (1.57–1.95)], disseminated cancer following LE reconstruction [aOR (CI) 3.58 (2.324–5.53)], and history of cardiovascular accident or stroke following breast reconstruction [aOR (CI) 16.97 (2.72–105.82)].

**Conclusion:**

Longer operating time was a significant predictor of SSI regardless of reconstruction site. Reducing operating times through proper surgical planning might help mitigate the risk of SSI following RFS. Our findings should be used to guide patient selection, counseling, and surgical planning prior to RFS.

## Introduction

Reconstructive flap surgery (RFS) is defined as the process of harvesting and transferring skin, fat, and/or muscle tissue from one area of the body to another. This tissue can either be displaced to the new area while maintaining its original vasculature, in the form of a “pedicled flap”, or be entirely separated from its origin and re-attached to new vasculature at the recipient site, in the form of a “free flap” (1).

RFS has revolutionized the field of plastic and reconstructive surgery. It is commonly used for the purpose of repairing congenital defects or defects following infection, traumatic injuries, or cancer resection (2, 3). Potential complications arising from RFS include flap-specific complications, e.g., wound dehiscence, tissue rejection, scarring, hematoma, edema, blood clotting, fistulas, to name a few. Another possible complication of RFS is surgical site infection (SSI) (4, 5).

SSI is defined by the Centers for Disease Control and Prevention (CDC) as a surgery-related infection that occurs at or near the surgical incision within 30 days of surgery. An infection can involve the skin at the site of incision (superficial incisional SSI), the underlying tissue and muscle (deep incisional SSI) or spread further into the organs and/or space between the organs (organ space SSI). SSI is one of the most common nosocomial infections, accounting for about 20% of all hospital-acquired infections, increasing mortality rates by up to 11-fold as well as inflicting significant financial burden on the healthcare sector (6). SSI is a serious postoperative complication that can lead to increased hospital length of stay, delayed wound healing, impaired tissue repair and flap necrosis leading to graft failure (7, 8).

The incidence of SSI following RFS varies according to the flap recipient site. It has been reported to occur following 4.9% of breast reconstructive procedures and up to 16.5% of head and neck reconstructive procedures. Moreover, predictors of SSI following these procedures have been described in the literature. Active smoking and hypertension were found to be significant predictors of SSI following breast reconstruction, while increased length of hospital stay was a significant predictor of SSI following head and neck reconstruction (8, 9). Nonetheless, the incidence and predictors of SSI following trunk and extremity reconstruction have not been investigated.

Hence, we queried the American College of Surgeons National Surgical Quality Improvement Program (ACS NSQIP) database to compare the incidence and determine the predictors of SSI following RFS across reconstruction sites. We hypothesize that due to the different etiologies and surgical techniques employed in the different reconstruction sites, the incidence and predictors of SSI will differ across the various sites. We anticipate that this analysis will contribute to improving patient selection, preoperative counseling, and surgical planning in hopes of mitigating the risk of SSI and its consequences following RFS.

## Materials and methods

### Dataset

Our study is a retrospective analysis of prospectively collected clinical data within the NSQIP database from years 2005 to 2020. The NSQIP database contains data on over 150 variables, including preoperative demographics, comorbidities, lab values, operative characteristics, and intra- and postoperative outcomes. These variables are collected within 30 days of surgery in both the inpatient and outpatient settings. The NSQIP data come from more than 700 participant sites. At each site, data is collected by trained and certified surgical clinical reviewers (SCRs) using a variety of methods including medical chart abstraction. To ensure high quality of data collection, SCRs receive mandatory web-based training, annual certification exams, and the participating sites receive inter-rater reliability (IRR) audits by the ACS NSQIP (10).

### Patient selection

We queried the NSQIP dataset for patients who underwent RFS using current procedural terminology (CPT) codes pertaining to free or pedicled flaps (Supplementary Tables S1, S2). To standardize our patient population and minimize confounding, we only included patients who underwent flap surgery as a principal procedure. Patients who underwent RFS using free or pedicled flaps were included in our patient cohort. The included CPT codes, with their frequency and description, are summarized in Supplementary Table S1. We excluded from our analysis patients who underwent RFS involving grafts, skin flaps, or surgeries of unspecified location. The excluded CPT codes, with their frequency, description, and reason for exclusion, are summarized in Supplementary Table S2.

### Data cleaning and management

Among our patient cohort, *n *= 339 (0.9%) had missing height values which were replaced with the median height (65 inches), and *n *= 189 (0.5%) had missing weight values which were replaced with the median weight (174 pounds). The values of height and weight were used to calculate the body mass index (BMI) in kg/m^2^ of the whole population and subpopulations.

Due to the heterogeneity in reporting operating times in NSQIP, operating times <1st percentile (35 min) of *n *= 329 (0.88%) patients were replaced with the median operating time (272 min). Additionally, *n *= 178 (0.48) missing values of length of total hospital stay were replaced with the median length of total hospital stay (4 days), *n *= 772 (2.1%) missing values of white blood cell (WBC) count were replaced with the median WBC count (6,600), and *n *= 694 (1.9%) missing values of creatinine (Cr) level were replaced with the median Cr level (0.8). For categorial variables, *n *= 88 (0.2%) patients had missing American Society of Anesthesiologists (ASA) classifications which were replaced with the mode ASA class (II—mildly disturbed).

The primary outcome of interest in our study was the incidence of SSI in each reconstruction site. SSI was defined as any post-surgical infection including: “superficial incisional SSI”, “deep incisional SSI”, and “organ/space SSI”. The complete definitions of these variables are available in the User Guide for the ACS NSQIP Procedure Targeted Participant Use Data File (11). Furthermore, patients were stratified based on five sites of flap reconstruction: breast, trunk, head and neck (H&N), upper extremity (UE) and lower extremity (LE).

### Data analysis

Descriptive statistics were calculated for all the patients with RFS, and patients stratified by reconstruction sites. Univariate exploratory analysis was done for the whole population and subpopulations. We presented normally distributed numerical data as mean ± standard deviation (SD) and non-normally distributed data as median and interquartile range (IQR). We used the independent t-test to compare continuous variables, and the Chi-square or Fisher's exact tests to compare categorical variables.

For each of the five reconstruction sites, we performed bivariate analysis to identify demographics, clinical and surgical characteristics that significantly differed between patients who developed SSI compared to those who did not. These covariates included age, BMI, operating time, preoperative WBC count, preoperative Cr levels, gender, race, Hispanic or Latinx ethnicity, smoking with 12 months prior to surgery, alcohol use, diabetes mellitus, chronic obstructive pulmonary disease (COPD), hypertension, congestive heart failure (CHF) in 30 days prior to surgery, previous percutaneous intervention (PCI), previous cardiac surgery, history of revascularization for peripheral vascular disease (PVD), history of transient ischemic attack (TIA), history of cerebrovascular accident (CVA) or stroke, steroid use within 30 days prior to surgery, disseminated cancer, chemotherapy (CT) within 30 days prior to surgery, radiotherapy (RT) within 90 days prior to surgery, bleeding disorders, preoperative dyspnea, requiring ventilator-assisted respiration within 48 h prior to surgery, pneumonia, requiring dialysis within 2 weeks prior to surgery, more than 10% of total body weight loss within 6 months prior to surgery, transfusion >4 units in 72 h prior to surgery, prior operation within 30 days, functional health status, ASA classification, presence of open wound, and preoperative systemic inflammatory response syndrome (SIRS). Covariates found to have a *p*-value < .2 on bivariate analysis were subsequently included in five different multivariate regression models to determine the significant predictors of SSI in each reconstruction site. To determine the most parsimonious risk model of SSI in each reconstruction site, forward logistic regression was used. The strength of the association between risk factors and incidence of SSI was estimated using the adjusted odds ratio (aOR) and 95% confidence interval (CI). A *p*-value < .05 was deemed significant. IBM SPSS Statistics 28.0.0.0 was used for data cleaning, management, and analysis (12).

## Results

**Figure 1:**
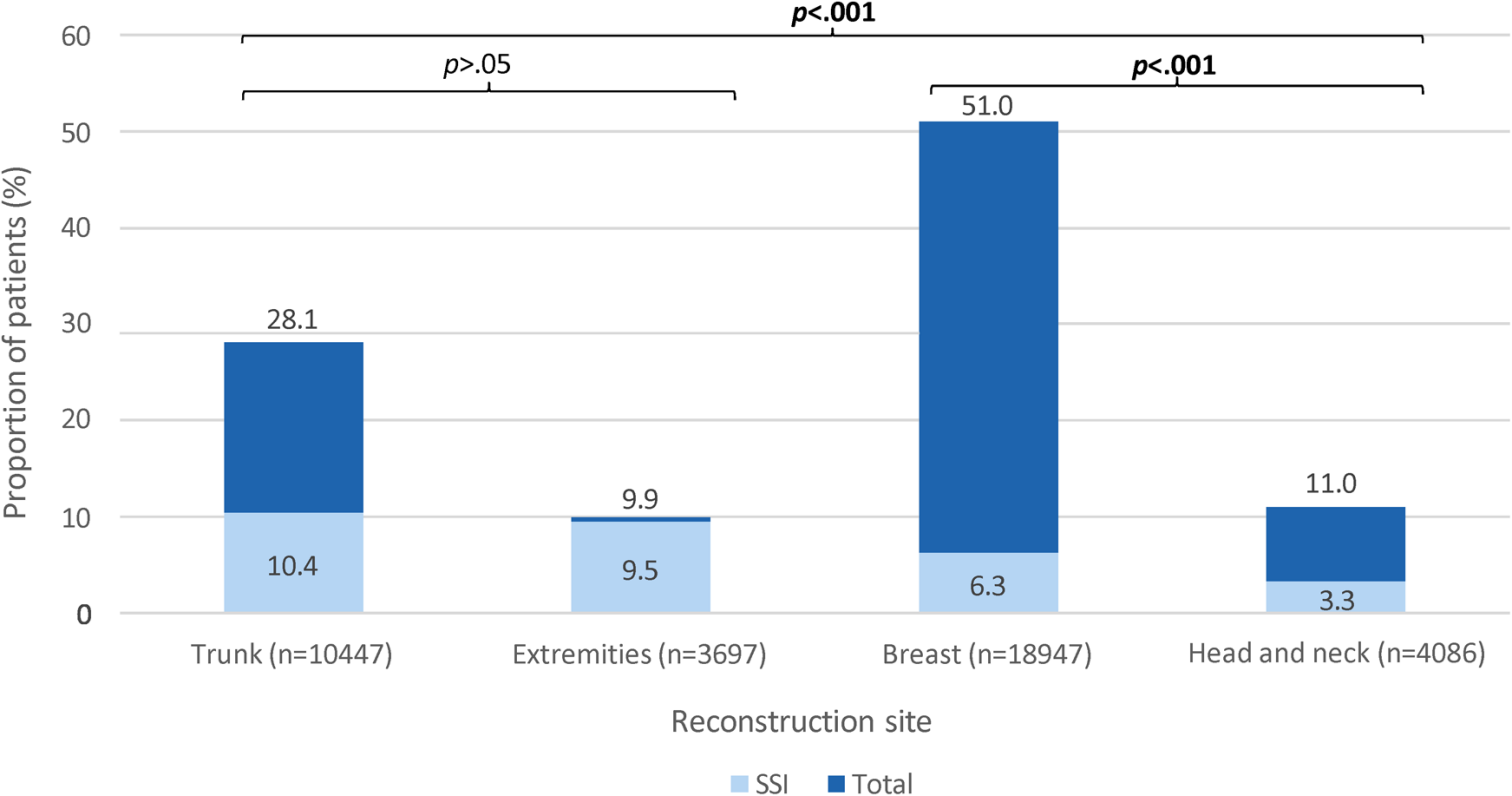
A stacked column chart showing the proportion of patients (%) who developed surgical site infection (light blue) among those who underwent reconstructive flap surgery (dark blue), stratified by reconstruction site. SSI, surgical site infection. Dark blue bars show the proportions of patients who underwent reconstructive flap surgery, stratified by reconstruction site. The proportions are calculated from the total number of patients who underwent reconstructive flap surgery (*n* = 37,177). Light blue bars show the proportions of patients who developed SSI following reconstructive flap surgery, stratified by reconstruction site. The proportions are calculated from the total number of patients who underwent reconstruction of each respective site (*x*-axis). These proportions are compared; significant *p*-values <.05 are bolded.

A total of 37,177 patients who underwent RFS were included in our patient cohort. Their mean age ± standard deviation (SD) was 54.76 ± 13.49 years, and their median (IQR) BMI was 28.8 (24.9–33.1) kg/m^2^. Median (IQR) total operating time was 272 (148–439) minutes, and median (IQR) length of total hospital stay was 3 (2–5) days. Figure 1 (dark blue bars) illustrates the distribution of patients who underwent RFS according to sites of reconstruction. The majority of patients in our patient cohort underwent breast reconstruction (*n *= 18,947, 51.0%), followed by trunk (*n *= 10,447, 28.1%), H&N (*n *= 4,086, 11.0%), LE (*n *= 2,963, 8.0%), and UE (*n *= 3,697, 9.92.0%) reconstruction (Figure 1—dark blue bars). A total of *n *= 2,776 (7.5%) patients developed SSI following RFS, the majority of whom had breast reconstruction (*n *= 1,201, 43.3%). Figure 1 (light blue bars) illustrates the proportion of patients who developed SSI within 30-days following RFS, stratified by reconstruction site. The proportion of patients who developed SSI following LE (*n *= 318, 10.7%) and trunk (*n *= 1,091, 10.4%) reconstruction was significantly greater than the proportion of patients who developed SSI following breast (*n *= 1,201, 6.3%), UE (*n *= 32, 4.4%), and H&N (*n *= 134, 3.3%) reconstruction (*p* < .001) (Figure 1—light blue bars). Table 1 compares patient demographics, clinical, and surgical characteristics between patients who developed SSI vs. those who did not develop SSI following RFS of each site.

**Table 1:** Demographics of SSI group versus non-SSI group stratified according to flap surgery.

Total (*n* = 2,776)	Breast			Trunk			H&N			LE			UE		
	+ SSI (*n* = 1,201)	− SSI (*n* = 17,746)	*p*-value	+ SSI (*n* = 1,091)	− SSI (*n* = 9,356)	*p*-value	+ SSI (*n* = 134)	− SSI (*n* = 3,952)	*p*-value	+ SSI (*n* = 350)	− SSI (*n* = 3,347)	*p*-value	+ SSI (*n* = 31)	− SSI (*n* = 279)	*p*-value
Age in years (mean ± SD)	51.56 ± 9.98	51.56 ± 9.85	0.983	56.07 ± 13.97	56.54 ± 14.92	0.304	61.58 ± 14.81	63.7 ± 15.63	0.125	58.36 ± 16.4	56.7 ± 16.8	0.092	53.53 ± 19.6	55.2 ± 18	0.542
BMI in kg/m^2^ (median; IQR)	31.24; 27.6–35.3	28.7; 25.2–32.6	**<** **.** **001**	31.34; 25.79–37.05	29.5; 25.07–34.5	**<** **.** **001**	26.38; 24.00–32.58	27.08; 23.54–31.29	0.785	24.40; 24.2–33.4	28.1; 24.1–32.3	0.317	27.5; 22.6–30.4	27.5; 24–32	0.371
WBC (median; IQR)	6.6; 5.3–7.3	6.5; 5.1–7	**<** **.** **001**	7.1; 6.3–8.9	6.6; 6.1–8.5	**<** **.** **001**	6.6; 6.32–8.56	6.6; 6.6–7.6	0.084	6.9; 6.2–8.8	6.9; 6.1–8.7	0.758	6.7; 6.5–9.0	6.6; 6.5–8.1	0.227
Creatinine (median; IQR)	0.8; 0.7–0.8	0.8; 0.7–0.8	0.250	0.8; 0.7–1	0.8; 0.7–1	0.177	0.8; 0.7–1.1	0.8; 0.8–0.97	0.161	0.8; 0.7–1.0	0.8; 0.7–1.0	0.667	0.8; 0.7–0.9	0.8; 0.8–0.9	0.589
Male gender	0.10%	0.20%	0.727	48.30%	51.10%	0.076	57.5%	57.3%	1.000	56.00%	57.40%	0.630	62.50%	58.00%	0.578
Race			**<** **.** **001**			0.955			**0** **.** **003**			0.310			0.338
White	64.80%	64.00%		78.30%	78.70%		73.1%	85.3%		75.80%	75.30%		74.20%	75.30%	
American Indian or Alaska Native	0.40%	0.30%		0.70%	0.50%		0.0%	0.3%		0.90%	1.00%		0%	0.60%	
Asian	3.60%	1.20%		1.40%	1.30%		0.7%	1.0%		0.90%	1.90%		6.50%	1.80%	
Black or African	13.10%	11.40%		10.80%	10.60%		4.5%	3.7%		9.10%	11.20%		3.20%	8.50%	
American Native Hawaiian or Pacific Islander	0.20%	0.30%		0.10%	0.10%		0.0%	0.1%		0.30%	0.10%		0%	0.10%	
Unknown/Not Reported	18.80%	22.00%		8.70%	8.70%		21.6%	9.6%		12.90%	10.60%		16.10%	13.70%	
Hispanic/Latinx ethnicity	6.70%	7.30%	**.** **008**	6.80%	6.70%	0.992	0.7%	3.4%	**<** **.** **001**	5.30%	6.00%	0.237	6.50%	5.30%	0.869
Smoker	11.80%	7.70%	**<** **.** **001**	25.50%	19.00%	**<** **.** **001**	23.9%	21.0%	.413	23.60%	22.70%	0.568	35.50%	25.60%	0.294
Alcohol	73.10%	68.20%	**<** **.** **001**	65.40%	67.70%	0.134	58.2%	57.9%	1.000	69.80%	67.30%	0.375	77.40%	67.90%	0.326
Diabetes Mellitus			**<** **.** **001**			**<** **.** **001**			.734			0.407			0.789
Insulin	3.60%	1.50%		12.50%	8.30%		6.00%	4.90%		9.40%	8.60%		6.30%	5.60%	
Oral agent	7.70%	5.10%		11.10%	10.40%		10.40%	9.20%		11.00%	9.00%		9.40%	8.40%	
COPD	1.30%	0.60%	**.** **003**	8.50%	5.80%	**<** **.** **001**	6.70%	5.30%	0.487	7.50%	4.80%	**.** **039**	12.90%	5.30%	0.090
Hypertension	31.80%	25.10%	**<** **.** **001**	51.10%	46.10%	**.** **002**	55.2%	51.1%	.352	51.60%	46.20%	0.074	29.00%	40.80%	0.261
CHF in 30 days prior to surgery	0.10%	0.10%	0.481	0.90%	1.20%	0.399	1.5%	0.70%	.234	1.30%	1.20%	1.000	0%	0.60%	1.000
Previous PCI	–	–	–	0.5%	0.6%	0.539	1.50%	0.50%	0.138	0.30%	0.60%	1.000	–	–	–
Previous cardiac surgery	0.20%	0.00%	0.068	0.80%	0.90%	0.706	0.70%	0.70%	1.000	0.90%	0.60%	0.432	–	–	–
History of revascularization for PVD	–	–	–	0.40%	0.30%	0.529	0.00%	0.30%	1.000	0.60%	0.80%	1.000	–	–	–
History of TIA	0.00%	0.10%	1.000	0.0%	0.10%	0.387	0.0%	0.3%	1.000	0.0%	0.30%	1.000	0%	0.10%	1.000
History of CVA/Stroke	0.20%	0.00%	0.035	0.50%	0.20%	0.142	0.0%	0.2%	1.000	0.30%	0.10%	0.365	0%	0.30%	1.000
Steroid use	2.20%	1.60%	0.069	5.40%	5.00%	0.52	8.2%	4.9%	.082	5.00%	4.30%	0.561	9.70%	5.20%	0.227
Disseminated cancer	1.40%	1.30%	0.776	4.50%	3.90%	0.326	6.7%	2.1%	**.** **003**	10.10%	2.90%	**<0** **.** **001**	0%	1.50%	1.000
CT in 30 days pre-op	0.30%	0.50%	0.660	0.20%	0.20%	1.000	0.0%	0.2%	1.000	0.30%	0.10%	0.365	–	–	–
RT in 90 days pre-op	0.00%	0.10%	1.000	0.10%	0.10%	1.000	0.0%	0.1%	1.000	0.00%	0.20%	1.000	–	–	–
Bleeding disorders	0.70%	0.70%	0.931	5.20%	4.40%	0.215	4.5%	3.9%	.757	6.60%	7.00%	0.815	3.10	3.00%	1.000
Dyspnea			**<** **.** **001**			**<** **.** **001**			.474			0.106			**.** **014**
Moderate exertion	2.60%	1.50%		10.40%	6.90%		9.0%	6.6%		7.20%	4.50%		16.10%	4.70%	
At rest	0.30%	0.00%		1.00%	0.80%		0.0%	0.2%		0.60%	0.80%		3.20%	0.90%	
Ventilator use	0.10%	0.00%	0.063	0.50%	0.90%	0.142	0.0%	0.3%	1.000	0.00%	0.60%	0.404	0%	0.30%	1.000
Pneumonia	–	–	**-**	0.0%	0.1%	1.000	0.0%	0.1%	1.000	–	–	–	–	–	–
On dialysis	0.20%	0.00%	0.129	1.20%	1.40%	0.613	2.2%	0.4%	**.** **023**	0.90%	2.20%	0.149	3.20%	3.40%	1.000
>10% body weight loss	0.60%	0.20%	**0** **.** **012**	3.90%	2.20%	**<** **.** **001**	5.2%	1.5%	**.** **006**	3.10%	1.70%	0.139	3.20%	1.00%	0.302
Transfusion >4U in 72 h preoperatively	0.10%	0.10%	0.600	1.40%	1.20%	0.704	0.7%	0.2%	.23	2.60%	2.40%	0.859	3.2%	1.20%	0.332
Prior operation within 30 days	0.50%	0.20%	**0** **.** **035**	0.90%	0.90%	1.000	1.5%	0.7%	.234	0.30%	1.80%	**.** **033**	0%	0.60%	1.000
Functional health status			**0** **.** **034**			**0** **.** **016**			**.** **007**			0.795			0.226
Independent	99.80%	99.00%		89.60%	91.60%		91.0%	96.3%		88.10%	85.70%		90.60%	94.30%	
Partially dependent	0.20%	0.60%		7.80%	5.70%		6.7%	2.5%		9.40%	10.70%		6.30%	4.30%	
Totally dependent	0.00%	0.00%		1.70%	2.10%		2.2%	0.7%		1.90%	2.60%		3.10%	0.90%	
Unknown	0.10%	0.30%		0.90%	0.50%		0.0%	0.6%		0.60%	0.90%		0.00%	0.60%	
ASA classification			**0** **.** **031**			**<** **.** **001**			**<** **.** **001**			0.480			**.** **058**
I	3.00%	3.60%		1.70%	3.20%		1.5%	4.6%		3.20%	4.40%		16.10%	8.30%	
II	58.70%	61.50%		26.70%	33.80%		27.6%	42.1%		32.00%	37.20%		25.80%	44.60%	
III	37.60%	34.50%		64.60%	56.20%		61.9%	49.0%		56.50%	50.9%		51.60%	44.42%	
IV	0.70%	0.30%		7.00%	6.90%		9.0%	4.3%		8.30%	6.70%		6.70%	2.90%	
V	–	–		–	–		0.0%	0.1%		0.00%	0.1%		–	–	
Open wound	2.80%	1.30%	**<** **.** **001**	30.10%	21.90%	**<** **.** **001**	29.1%	19.2%	**.** **004**	45.60%	45.00%	0.858	38.7%	25.8%	0.142
SIRS	0.20%	0.30%	0.769	5.60%	3.40%	**<** **.** **001**	4.5%	0.6%	**<** **.** **001**	6.80%	4.30%	0.059	0.14%	0.1%	1.000
Operating time in minutes (median; IQR)	442; 314–566	404; 279–536	**<** **.** **001**	212; 139–320	176; 113–272	**<** **.** **001**	196.5; 121.5–346.3	131; 81–220	**<** **.** **001**	169 (108, 247)	141 (87, 226)	**<** **.** **001**	148 (104–275)	115 (67–212)	**.** **048**

H&N, head and neck; UE&LE, upper extremity and lower extremity; SSI, surgical site infection; BMI, body mass index; WBC, white blood cell; COPD, chronic obstructive pulmonary disease; CHF, congestive heart failure; PCI, percutaneous coronary intervention; PVD, peripheral vascular disease; TIA, transient ischemic attack; CVA, cerebrovascular accident; CT, chemotherapy; RT, radiotherapy; ASA, American Society of Anesthesiologists; SIRS, systemic inflammatory response syndrome; SD, standard deviation; IQR, interquartile range. Significant *p*-values are bolded.

Variables with a *p *< .2 upon comparison of patients’ demographics (Table 1) were used in multivariate logistic regression models (Table 2) to assess the significant predictors of SSI following RFS of each site. After adjustment with multivariate logistic regression, longer operating time was a significant predictor of SSI following RFS across all sites, and the presence of open wound was a significant predictor of SSI following RFS across most reconstruction sites (except extremities).

**Table 2:** Multivariate regression evaluating predictors of SSI following flap reconstruction stratified by flap reconstruction site.

Risk factors	Breast		Trunk		H&N		LE		UE	
	aOR	95% CI	aOR	95% CI	aOR	95% CI	aOR	95% CI	aOR	95% CI
BMI in kg/m^2^ (median; IQR)	1.06	**1** **.** **05–1** **.** **07**	1.03	**1** **.** **02–1** **.** **04**	–	–	–	–	–	–
WBC count	–	–	–	–	1.04	**1** **.** **02–1** **.** **05**	–	–	–	–
Male gender	–	–	0.86	0.76–0.98	–	–	–	–	–	–
**Race**										
American Indian or Alaska Native	Ref	–	–	–	Ref	–	–	–	–	–
Asian	0.54	0.17–1.71	–	–	0.48	0.26–0.91	–	–	–	–
Black or African American	0.80	0.28–2.24	–	–	0.79	0.46–1.37	–	–	–	–
Native Hawaiian or Pacific Islander	2.50	0.58–10.78	–	–	1.26	0.46–3.45	–	–	–	–
White	1.27	0.46–3.53	–	–	1.02	0.6–1.76	–	–	–	–
Unknown/Not Reported	1.29	0.45–3.66	–	–	0.88	0.51–1.49	–	–	–	–
**Hispanic ethnicity**										
No	Ref	–	–	–	–	–	–	–	–	–
Yes	0.88	0.69–1.13	–	–	–	–	–	–	–	–
Unknown	1.33	**1** **.** **04–1** **.** **71**	–	–	–	–	–	–	–	–
Smoker	1.64	**1** **.** **36–1** **.** **98**	1.43	**1** **.** **23–1** **.** **66**	–	–	–	–	–	–
Alcohol	1.27	**1** **.** **11–1** **.** **45**	–	–	–	–	–	–	–	–
**Diabetes mellitus**										
No	Ref	–	–	–	–	–	–	–	–	–
Insulin	2.00	**1** **.** **42–2** **.** **82**	–	–	–	–	–	–	–	–
Oral agent	1.25	0.98–1.60	–	–	–	–	–	–	–	–
COPD	–	–	–	–	–	–	–	–	–	–
Hypertension	1.21	**1** **.** **06–1** **.** **39**	–	–	–	–	1.28	**1** **.** **01–1** **.** **62**	–	–
Previous cardiac surgery	–	–	–	–	–	–	–	–	–	–
History of CVA/Stroke	16.97	**2** **.** **72–105** **.** **82**	–	–	–	–	–	–	–	–
Disseminated cancer	–	–	–	–	1.56	**1** **.** **27–1** **.** **93**	3.58	**2** **.** **32–5** **.** **54**	–	–
**Dyspnea**										
No	Ref	–	Ref	–	–	–	–	–	Ref	–
Moderate exertion	1.32	0.90–1.95	1.36	**1** **.** **09–1** **.** **69**	–	–	–	–	4.2	**1** **.** **47–12**
At rest	5.13	**1** **.** **42–18** **.** **52**	1	0.52–1.92	–	–	–	–	5.7	0.64–49.6
Ventilator dependent	>100	–	0.31	**0** **.** **12–0** **.** **79**	–	–	–	–	–	–
>10% body weight loss	4.11	**1** **.** **78–9** **.** **49**	1.58	**1** **.** **11–2** **.** **24**	1.67	**1** **.** **27–2** **.** **19**	–	–	–	–
Prior operation within 30 days	3.50	**1** **.** **44–8** **.** **49**	–	–	–	–	0.16	0.02–1.16	–	–
Open wound	2.36	**1** **.** **62–3** **.** **45**	1.82	**1** **.** **57–2** **.** **11**	1.75	**1** **.** **57–1** **.** **95**	1.29	**1** **.** **03–1** **.** **61**	–	–
SIRS	–	–	1.59	**1** **.** **18–2** **.** **14**	1.51	**1** **.** **18–1** **.** **93**	–	–	–	–
Operating time in minutes (median; IQR)	1.001	**1** **.** **001–1** **.** **001**	1.002	**1** **.** **002–1** **.** **003**	1.001	**1** **.** **001–1** **.** **001**	1.002	**1** **.** **001–** **.** **002**	**1** **.** **003**	**1** **.** **001–1** **.** **005**
**ASA class**										
I	–	–	–	–	Ref	–	–	–	–	–
II	–	–	–	–	1.18	0.92–1.51	–	–	–	–
III	–	–	–	–	1.53	**1** **.** **2–1** **.** **96**	–	–	–	–
IV	–	–	–	–	1.45	**1** **.** **06–1** **.** **98**	–	–	–	–
V	–	–	–	–	0.0	0.0	–	–	–	–
C-statistic	0.66	0.66	0.61	0.60	0.67
Hosmer-Lemeshow test	0.33	0.05	0.15	0.09	0.007

H&N, head and neck; UE & LE, upper extremity and lower extremity; BMI, body mass index; SIRS, systemic inflammatory response syndrome; aOR, adjusted odds ratio; CI, 95% confidence interval; IQR, interquartile range; Ref, reference category; C-statistic, concordance statistic or the area under the receiver operating curve. Significant 95% confidence intervals are bolded.

The strongest predictor of SSI following breast reconstruction was history of CVA/stroke [adjusted odds ratio (aOR) 95% confidence interval (CI) 16.97 (2.72–105.82)] followed by preoperative dyspnea at rest [aOR (CI) 5.13 (1.42–18.52)]. Alcohol use [aOR (CI) 1.27 (1.11–1.45)] and diabetes mellitus on insulin [aOR (CI) 2.0 (1.42–2.82)] were unique risk factors for SSI following breast reconstruction. Other risk factors included BMI, smoking, hypertension, loss of more than 10% of body weight within 6 months prior to surgery, and prior operation within 30 days (Table 2). The presence of open wound was the strongest predictor of SSI following trunk reconstruction [aOR (CI) 1.82 (1.57–2.11)], followed by preoperative SIRS [aOR (CI) 1.59 (1.18–2.14)]. In our analysis, requiring ventilator-assisted respirations within 48 h prior to surgery was a unique and protective factor for SSI following trunk reconstruction [aOR (CI) 0.31 (0.12–0.79)]. Other risk factors included BMI, smoking, loss of more than 10% of body weight within 6 months prior to surgery, and preoperative dyspnea 226,201 with moderate exertion (Table 2). Similarly, the presence of open wound was also the strongest predictor of SSI following H&N reconstruction [aOR (CI) 1.75 (1.57–1.95)], followed by loss of more than 10% of body weight within 6 months prior to surgery [aOR (CI) 1.67 (1.27–2.19)] and disseminated cancer [aOR (CI) 1.56 (1.27–1.93)]. Preoperative WBC count was a unique risk factor for SSI following H&N reconstruction [aOR (CI) 1.04 (1.02–1.05)]. Other risk factors included preoperative SIRS, ASA class III and ASA class IV (Table 2). As for extremity reconstruction, the strongest predictor of SSI was disseminated cancer [aOR (CI) 3.58 (2.324–5.543)] following LE reconstruction, and preoperative dyspnea at rest [aOR (CI) 4.2 (1.47–12)] following UE reconstruction.

## Discussion

Postoperative SSI is a serious and unfortunate complication of RFS leading to morbidity and mortality (6, 7). Using NSQIP, a multi-institutional database, we conducted the first and largest study to date to compare the incidence and determine the predictors of SSI following RFS across reconstruction sites. Our starting hypothesis was that both the incidence and predictors will vary across reconstruction sites due to the different reasons for and techniques of RFS at the different sites. We aim to provide surgeons with conclusions that can help guide patient selection, counseling, and surgical planning to help minimize the risk of SSI postoperatively. The overall 30-day incidence rate of SSI in our patient cohort was 7.5%, which falls within the range reported in the literature (8, 9, 13). However, due to different techniques and sites of reconstruction, surgeon expertise, patient demographics and comorbidities, and duration of patient follow-up, accurate complication rates may be difficult to compare among studies (14). In the subpopulation of patients who underwent breast RFS, 6.3% of patients developed SSI. This incidence rate was within the range reported in the literature (15). Our results showed that patients with higher BMI, smoking, hypertension, and diabetes mellitus were at significantly greater odds of SSI following breast RFS. Similarly, multi-institutional studies using NSQIP on patients who underwent breast reconstruction reported obesity, smoking, hypertension, and diabetes mellitus to be associated with increased odds of wound complications, such as SSI within 30 days of surgery (9, 16, 17). Obesity has been hypothesized to contribute to an increased occurrence of SSI through decreased oxygen supply, impaired penetration of prophylactic antibiotics, longer operating times, increased blood loss perioperatively, and impaired immunity (18–22). Smoking and hypertension have been found to contribute to the risk of SSI due to reduced tissue perfusion and delayed healing (23, 24). Similarly, diabetes might contribute to the risk of SSI through vascular changes and impaired immunity (25, 26). Some other risk factors of SSI in our RFS of the breast cohort, not previously reported in the literature, include longer operating time, presence of open wound, and history of CVA/stroke. According to our results, the latter was the strongest predictor of SSI following RFS of the breast. This can be explained by the tight association between stroke and the comorbidities previously discussed, including obesity, hypertension, and diabetes mellitus (27). Our results, in line with existing literature, highlight the importance of patient counseling about the importance of smoking cessation and proper management of patient comorbidities to mitigate the incidence of postoperative SSI.

In our patient cohort, the incidence rate of SSI following trunk reconstruction was lower than the range reported in the literature (20%–28.6%) (28, 29). According to our results, significant predictors of SSI following RFS of the trunk were obesity, smoking, presence of open wound, longer operating times, preoperative SIRS, loss of more than 10% of body weight within 6 months prior to surgery, and preoperative dyspnea with moderate exertion. Patient-related risk factors for SSI following trunk reconstruction have not been investigated before. However, the failure of removal of spine implants before RFS of the trunk has been reported to be associated with SSI (29).

For our H&N population consisting of 2,381 patient who underwent RFS, 4.2% of them (100 patients) developed SSI, which is in discordance with the range reported in existing literature (7, 8, 30, 31). In a study by Lebo et al. on 4,014 patients with complex H&N surgery, 16.5% of them (662 patients) developed SSI within 30 days of surgery. They showed that smoking, diabetes, prior wound infection, contaminated or dirty wound classes, and longer operating times were 264,238 risk factors for postoperative SSI (8). Among those risk factors, only longer operating time was found to be a significant predictor of SSI following RFS of the H&N in our analysis. The difference in incidence and predictors of SSI between our study and the one by Lebo et al. may be attributed to the different inclusion/exclusion criteria of both studies. For instance, Lebo et al. did not include patients who underwent flap reconstruction of the H&N using muscle, myocutaneous or fasciocutaneous flaps (CPT code: 15732). Additionally, they included patients with laryngectomy (CPT codes: 31360, 31365, 31368, 31390, 31395), which we did not include, and patients with reconstruction of unspecified site (CPT codes: 15756, 15758), skin flaps (CPT code: 15757), and bone grafts (CPT codes: 20955, 20956) which we excluded (8). Additional risk factors for SSI following RFS of the H&N in our analysis were longer operating times, presence of open wound (aOR = 1.75), loss of more than 10% of body weight within 6 months prior to surgery, disseminated cancer, high preoperative WBC count, preoperative SIRS, ASA class III and ASA class IV. Patients with H&N cancer who have disseminated disease typically undergo neoadjuvant chemotherapy and radiotherapy before surgery, so they may present at the time of surgery with low levels of white blood cells. Thus, the significant association between WBC count and SSI may not be a causal relationship. Overall, risk factors of SSI following trunk and H&N reconstruction are reflective of deteriorating/poor health. These results can be beneficial to surgeons for proper informed decision making.

As for extremity reconstruction, the incidence of SSI following RFS in either extremity was lower than the one reported in the literature by Arakelyant et al. (27.5%) who analyzed the extremities combined (32). In our analysis, we have separated patients who performed RFS of either extremity due to the different etiologies of RFS and the different potential risk factors that might lead to SSI in each. Patient-related risk factors of SSI following UE&LE reconstruction have not been investigated previously. However, external protective factors against SSI have been reported and include early wound cleaning and reconstruction, the use of negative pressure wound therapy before reconstruction, and free flaps rather than local flaps (33–36). In patients who had RFS of the LE, the strongest predictor of SSI was disseminated cancer, a logical reason for RFS of the LE, and a surrogate for overall impaired systemic immunity (37), which potentially increases the risk of postoperative SSI. Patients who had developed SSI following UE reconstruction had increased odds of preoperative dyspnea [aOR (CI) 4.2 (1.47–12)]. Although the association between preoperative dyspnea and SSI is not clearly understood, preoperative dyspnea has been reported to be a risk factor of SSI in several studies (38–40).

Operating time has been perceived to be a surrogate for surgeons’ experience/number, with shorter operating times reflecting greater experience/number of the surgeon(s) performing the surgery. In a study by Gösseringer et al. on 14 patients, operating time decreased as number of performing surgeons increased, and was also paralleled with a decrease in the incidence of flap failure following breast reconstruction (41). Similarly, longer operating time in our study was significantly associated with 255 increased odds of SSI following RFS regardless of the site of reconstruction. Although we could not link this finding to surgeons’ experience/number, as these variables are not collected by NSQIP, longer operating time per say might put patients at increased risk of SSI and might be a surrogate for decreased experience/number of performing surgeon(s). However, it is possible that experienced surgeons often perform complex cases that require longer operating times. Due to this dilemma and the existing conflicting evidence (41, 42), further research and larger studies are needed to better determine the association between operating time and surgeons’ experience/number in RFS. Nonetheless, despite the ambiguity regarding contributors to operating time, proper surgical planning to reduce operating time might help mitigate the risk of SSI following RFS. In light of the retrospective nature of our study and the use of the NSQIP database, several limitations exist. First, there is a limited follow up as NSQIP database reports outcomes up to 30 days postoperatively. While this may underestimate the overall postoperative rate of SSI, this limitation of NSQIP is less pronounced in our case since most SSI cases would likely appear within 30 days of surgery. Second, NSQIP does not report patients/procedures equally across surgeons or centers, so the data might not be representative and might be subject to selection bias. Third, NSQIP does not allow us to determine whether SSI develop at the donor or recipient site. However, we suspect that most SSI cases would have developed at the recipient flap site where microsurgery was performed. Fourth, some important variables for our analysis were missing including surgeons’ experience/number, as mentioned above, and the use of perioperative antibiotics, which might influence the occurrence of SSI. Fifth, patients undergoing RFS at different reconstructive sites differed in number and proportion, which might give more weight to results generated from analysis of larger subgroups. Nevertheless, despite the above limitations, this is the first and largest study to compare incidence and determine risk factors of SSI following RFS across reconstruction sites.

## Conclusion

This study compares the incidence and determines the predictors of SSI within 30 days following RFS. Longer operating time was a significant predictor of SSI regardless of reconstruction site. Proper surgical planning might reduce the risk of SSI following RFS. Proper patient counseling or strict preoperative nicotine testing, as well as proper management of comorbidities such as obesity, hypertension, and diabetes mellitus might also help mitigate the risk of SSI following RFS. Our findings are thus important for patient selection, counseling, and surgical planning prior to RFS. Future prospective studies are needed to better evaluate SSI predictors following RFS.

## Data Availability

The datasets presented in this study can be found in online repositories. The names of the repository/repositories and accession number(s) can be found below: ACS NSQIP PUF datafiles 2008 to 2020.
