# Supplementary material for: Predictors of surgical site infection following reconstructive flap surgery: A multi-institutional analysis of 37,177 patients
Source: Front Surg. 2023 Jan 30;10:1080143. doi: 10.3389/fsurg.2023.1080143 (PMC9923723; doi:10.3389/fsurg.2023.1080143)
Supplement: Supplementary file 1 [file Datasheet1.docx]

**Supplementary Table 1.** Overview of the included CPT codes, frequency, percentage, and description of each.

| **Included CPT codes** | **Frequency** | **Percent (%)** | **Description of code** |
| --- | --- | --- | --- |
| 15570 | 91 | .2 | Formation of direct or tubed pedicle, with or without transfer;  trunk |
| 15576 | 232 | .6 | Formation of direct or tubed pedicle, with or without transfer;  eyelids, nose, ears, lips or intraoral |
| 15731 | 1733 | 4.7 | Forehead flap with preservation of vascular pedicle |
| 15732 | 2135 | 5.7 | Muscle, myocutaneous or fasciocutaneous flap; head and neck |
| 15734 | 10366 | 27.9 | Muscle, myocutaneous or fasciocutaneous flap; trunk |
| 15736 | 710 | 1.9 | Muscle, myocutaneous or fasciocutaneous flap; upper extremity |
| 15738 | 2963 | 8.0 | Muscle, myocutaneous or fasciocutaneous flap; lower extremity |
| 19361 | 4380 | 11.8 | Breast reconstruction with latissimus dorsi flap, without  prosthetic implant |
| 19364 | 12511 | 33.7 | Breast reconstruction with free flap |
| 19367 | 1640 | 4.4 | Breast reconstruction with transverse rectus abdominis myocutaneous flap (TRAM), single pedicle, including closure of  donor site |
| 19368 | 416 | 1.1 | Breast reconstruction with transverse rectus abdominis  myocutaneous flap (TRAM), single pedicle, including closure of donor site |
| Total | 37177 | 100 |  |

Percentages were calculated from the total number of patients with included CPT codes (*n*=37177).

| **Excluded CPT codes** | **Frequency** | **Percent (%)** | **Description of code** | **Reason for exclusion** |
| --- | --- | --- | --- | --- |
| 15572 | 106 | 0.24 | Formation of direct or tubed pedicle, with or without transfer; scalp, arms or legs | Unspecified site |
| 15574 | 262 | 0.60 | Formation of direct or tubed pedicle, with or without transfer; forehead, cheeks, chin,  mouth, neck, axillae, genitalia, hands or feet |  |
| 15650 | 147 | 0.34 | Transfer, intermediate, of any pedicle flap  (e.g. abdomen to wrist, walking tube),  any location |  |
| 15756 | 1900 | 4.34 | Muscle, myocutaneous or fasciocutaneous  flap; site not specified |  |
| 15758 | 977 | 2.23 | Free fascial flap with microvascular  anastomosis |  |
| 15757 | 2214 | 5.06 | Free skin flap (microvascular transfer) | Skin flap |
| 20955 | 168 | 0.38 | Bone graft with microvascular anastomosis;  fibula | Graft |
| 20956 | 75 | 0.17 | Bone graft with microvascular anastomosis;  metatarsal |  |
| 15732^1^ | 88 | 0.2 | Muscle-skin graft, head and neck |  |
| 15734^1^ | 447 | 1.02 | Muscle-skin graft, trunk |  |
| 15736^1^ | 22 | 0.05 | Muscle-skin graft, arm |  |
| 15738^1^ | 169 | 0.39 | Muscle-skin graft, leg |  |
| Total | 6575 | 15.03 |  | |

**Supplementary Table 2.** Overview of the excluded CPT codes, frequency, percentage, and description of each.

Percentages were calculated from the total number of patients with CPT codes pertaining to flap surgeries

(*n*=43752).

^1^ Surgeries with these CPT codes were performed using both free flaps and grafts. Surgeries that were performed

using free flaps were included (Supplementary table 1) and those using grafts were excluded (Supplementary table

2).
